# Myopic decisions under negative emotions correlate with altered time perception

**DOI:** 10.3389/fpsyg.2015.00468

**Published:** 2015-04-17

**Authors:** Shuchen Guan, Lu Cheng, Ying Fan, Xianchun Li

**Affiliations:** Key Laboratory of Brain Functional Genomics, Ministry of Education, Shanghai Key Laboratory of Brain Functional Genomics, School of Psychology and Cognitive Science, East China Normal University, Shanghai, China

**Keywords:** emotion, inter-temporal choice, time perception, response inhibition, myopic behavior

## Abstract

Previous studies have obtained inconsistent findings about emotional influence on inter-temporal choice (IC). In the present study, we first examined the effect of temporary emotional priming induced by affective pictures in a trial-to-trial paradigm on IC. The results showed that negative priming resulted in much higher percentages of trials during which smaller-but-sooner reward (SS%) were chosen compared with positive and neutral priming. Next, we attempted to explore the possible mechanisms underlying such emotional effects. When participants performed a time reproduction task, mean reaction times in negative priming condition were significantly shorter than those in the other two emotional contexts, which indicated that negative emotional priming led to overestimation of time. Moreover, such overestimation was negatively correlated with performance in the IC task. In contrast, temporary changes of emotional contexts did not alter performances in a Go/NoGo task (including commission errors and omission errors). In sum, our present findings suggested that myopic decisions under negative emotions were associated with altered time perception but not response inhibition.

## Introduction

Many decisions require trade-offs between amount of reward and time of delivery (McClure et al., 2004), which refers to inter-temporal choice (IC). IC is widely involved in many fields, such as finance, health, education, and so on. The subjective values of delayed reward are known to be discounted as a function of delay time following a hyperbolic function, which is defined as inter-temporal discounting or delay discounting (Peters, 2011). Previous studies have shown that delay discounting can be affected by age (Green et al., 1994, 1996), cultural differences (Kim et al., 2012), and psychological and psychiatrical disorders including substance abusers (Kirby and Petry, 2004), patients with Parkinson’s disease (Milenkova et al., 2011) and pathological gamblers (Kraplin et al., 2014).

In everyday life, human beings and animals continually engage in emotionally-driven behaviors (Angrilli et al., 1997). Accumulating evidence has revealed that emotion can influence many cognitions, including attention (Brosch and Van Bavel, 2012), sensory perception (Asutay and Vastfjall, 2012), and memory (Rimmele et al., 2011). Several studies have already displayed that emotional experiences (such as joy or regret) after decisions could guide subsequent choices (Brosch et al., 2013). Moreover, emotional regulation (such as relax-focused regulation) decreased risky behaviors (Martin and Delgado, 2011). Recent evidence has also revealed an important role of emotional context in IC processes. Human beings often display much steeper discounting rates in the IC by a stable negative emotion induced by successive presentations of negative pictures (Augustine and Larsen, 2011) or a natural disaster (Li et al., 2011). These findings suggest that negative context leads to near-sighted behaviors.

However, a recent study demonstrated that temporary negative (i.e., fear) priming by facial expressions in a trial-to-trial paradigm resulted in higher percentages of larger-but-later (LL) choices compared with neutral and positive priming (Luo et al., 2014), which indicates that negative priming makes human beings far-sighted. Such inconsistent findings leave the exact interaction between emotion and IC to be uncovered. Thus, in the present study, we first examined the influence of temporary emotional experience induced by International Affective Picture System (IAPS) pictures by a trial-to-trial paradigm on the IC task (Task 1). Figuring out the exact interaction between emotion and IC may help improve decision making in healthy humans as well as patients with psychological and psychiatrical disorders due to impaired IC in substance abusers (Kirby and Petry, 2004), schizophrenics (Weatherly, 2012), patients with Parkinson’s disease (Milenkova et al., 2011), and pathological gamblers (Kraplin et al., 2014).

The cognitive mechanisms underlying emotional effects on IC are still an open question. In our present study, we made an attempt to elucidate possible role of time perception (Task 2) and response inhibition (Task 3) in the emotional effect on IC. Recently, a growing body of literature has indicated that time perception, the subjective experience of time, potentially moderates IC (Takahashi, 2005; Kim and Zauberman, 2009). Patients with depression and ADHD usually overestimate durations in time-estimation tasks, which means they experience the feelings of time slowing down (Wittmann and Paulus, 2008; Droit-Volet, 2013). Meanwhile, they are more impulsive in IC tasks (Takahashi et al., 2008; Demurie et al., 2013). Therefore, the longer time perception in these patients is probably associated with higher preference for immediate reward. Such a relationship has also been obtained in normal individuals (Noulhiane et al., 2007). Given that time perception could be altered by emotions induced by emotional sounds (Noulhiane et al., 2007), film (Droit-Volet et al., 2011) and facial expressions (Droit-Volet et al., 2004), it is reasonable to propose that emotional effects on IC could be attributed to altered time perception by emotional context. Thus, we designed Task 2 to examine this hypothesis. After inducing emotional states in the same way as in Task 1, we evaluated individual’s time perception in a time reproduction task in different emotional contexts and examined the relationship between altered time perception and performance on IC.

Response inhibition is an inhibitory control to restrain the tendency to react to stimuli in a rapid and unplanned fashion without complete processing of information. Previous evidence has shown that cocaine-dependent patients showed much higher impulsivities by self-report measures, meanwhile they discounted delayed reward in a steeper rate compared with controlled ones (Coffey et al., 2003). PGs exhibited many more impairments in response inhibitions in a Go/NoGo task (Billieux et al., 2012) and preferred immediate, smaller reward (Dixon et al., 2003; Power et al., 2012; Kraplin et al., 2014). In addition, the impulsive individuals often discount reward more steeply than self-controlled ones (Wittmann and Paulus, 2008). On the other hand, a previous study revealed that there existed an interaction between emotion and response inhibition, for example, commission errors were more numerous and responses were slower in an emotional Go/NoGo task than in a non-emotional Go/NoGo task (Schulz et al., 2007). In Task 3 of the present study, we therefore asked participants to conduct a Go/NoGo task under different emotional contexts and investigated whether emotional effects on IC were related to altered response inhibition.

## Materials and Methods

### Participants

Twenty-seven college students (age: 19.3 ± 1.7 years; 20 females) were recruited in this study, one of them quitted while performing the task, so our data analyses were conducted on 26 participants. All of them had never performed similar experiments before. The study was approved by the University Committee on Human Research Protection in East China Normal University. An informed consent was obtained from each participant. After the experiment, participants received monetary reward or gained academic credits.

### Affective Stimuli

The affective pictures were selected from the IAPS (Lang et al., 2005). There were 50 pictures per type of stimuli, including negative (i.e., fear), positive (i.e., happy), and neutral. Then, 15 participants who did not participate in the formal experiment (age: 18.1 ± 1.0 years; 7 males) were required to evaluate the valence and arousal of the 150 pictures by a Likert-type scale (Lucci, 2013) with scores ranged from 1 to 9. The more negative the pictures are, the lower scores gained. Eighty-one stimuli (27 for each emotion valence) with the mean valence with smaller 3 (negative picture), 4.5–5.5 (neutral picture), or larger than 7 (positive picture) were used in the following formal experiment. The numbers for negative pictures were 3301, 2800, 3350, 9007, 9412, 3550, 9940, 9429, 6022, 3266, 6313, 6263, 6550, 2750, 9500, 6370, 3530, 9911, 2799, 9040, 6212, 2717, 3230, 6311, 6415, 6520, and 6350. The numbers for neutral pictures were 5535, 7041, 7021, 7000, 7011, 7006, 7025, 7042, 7590, 2383, 2377, 7033, 7045, 7044, 7595, 7211, 7061, 7002, 7003, 7004, 1903, 2382, 2411, 7059, 7010, 7009, and 7080. The numbers for positive pictures were 2274, 2311, 2530, 1441, 2154, 2345, 2070, 1750, 2045, 2091, 8600, 2387, 8330, 2158, 2040, 2347, 7325, 2035, 1340, 7502, 2057, 2360, 2071, 2050, 8497, 2165, and 2209. These stimuli were presented in the same size (size: 11.9 cm × 9 cm; view angle: 7°, presented in black background) at the center of a computer screen.

### Discounting Rate Estimation Procedures

This procedures were used to estimate individuals’ discounting rate before formal experiment according to the study by Kirby et al. (1999). Briefly, an estimate discounting rate parameter (k) for each participant could be made from his/her pattern of choices across 27 questions on the monetary-choice questionnaire which were randomly presented. The discounting rate was derived by the geometric mean of two k-values when participants just switched smaller-but-sooner (SS) gains to LL gains or the other way. For example, a participant chose the SS gain on the trial 24 offering a choice between “¥33 today” and “¥80 in 14 days” and chose the LL gain on the trial 27 offering a choice between “¥31 today” and “¥85 in 7 days.” Therefore, the discounting rate of this participant would be the geometric mean of the interval between 0.10 and 0.25 (see details in Kirby et al., 1999), it was 0.16.

Because of the magnitude effect during the delay discounting task (Hayashi et al., 2013), three levels of amount were used (Small: 25∼35; Medial: 50∼60; Large: 75∼85) in present study. After obtaining the three different k values, we adjusted the amounts of SS reward by 5% of up/down regulating k values by the following hyperbolic discount function: V = A/(1 + kD). Where V = time discounted value (i.e., “present value”) of a delayed amount; A = delayed amount, D = length of delay (in days) and k = the discounting rate (Mazur, 1987).

### Experimental Procedures

Each participant had to perform an IC task, a time reproduction task and a Go/NoGo task in current study. The order of the time reproduction task and the Go/NoGo task was counterbalanced across participants. All tasks were programmed in the E-Prime 2.0 (Psychology Software Tools inc. Pittsburgh, PA, USA).

#### Inter-Temporal Choice Task (Figure 1A)

**FIGURE 1:**
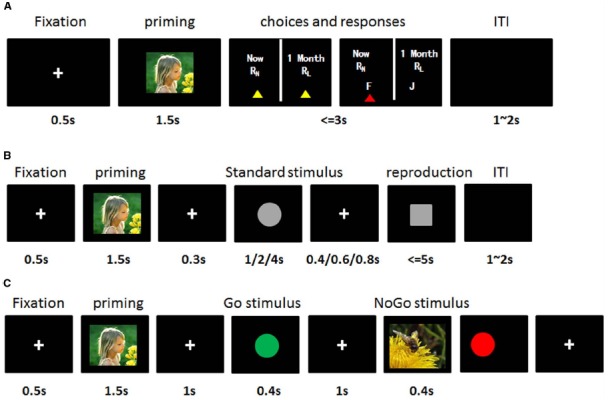
**The stimulus sequences and experimental design. (A)** Inter-temporal choice task. A fixation was displayed at the center of screen, which followed by an IAPS picture for 1.5 s. Then, participants should make their choices between a smaller-but-sooner (SS) reward and a larger-but-later (LL) reward within 3 s by key-pressing of “F” for SS or “J” for LL. R_N_ represents SS reward, RL indicates LL reward. **(B)** Time reproduction task: A fixation was displayed at the center of screen, which followed by an IAPS picture for 1.5 s. After a black screen for 0.3 s, a cycle stimulus as the standard stimulus (duration of 1,2, or 4 s) was presented which was followed by another black screen with duration of 0.4, 0.6, or 0.8 s. Then, the square stimulus appeared as a target, subjects were asked to press “F” in the keyboard (e.g., “F” key) as soon as they thought the duration of target stimuli was the same with standard stimulus. **(C)** Go/NoGo task: A fixation was displayed at the center of screen, which followed by an IAPS picture for 1.5 s. Then, either Go stimulus (e.g., green circle. Frequency, 70%) or NoGo stimulus (e.g., red circle. Frequency, 30%) was presented with duration of 0.4 s. Participants were asked to respond to either a Go stimulus by pressing “F” in the keyboard as soon as possible or a NoGo stimulus by withholding their response.

Participants sat approximately 50 cm from the screen (resolution: 1280 × 800). They were then instructed to imagine a situation that they actually faced and had to make a binary choice between a SS and a LL reward based on their own experience. Each trial started with a fixation (“+” in white on a black background; view angle: 0.8° × 0.8°) for 0.5 s. Then, one affective picture with negative, positive or neutral valence was presented on the screen for 1.5 s. Participants were required to comprehend the content of the picture. After the disappearance of the affective picture, SS and LL choices were presented at the same time. Participants were asked to press “F” on the keyboard if they preferred SS gains within 3 s, otherwise they should press “J” if they preferred LL gains. After button pressing, an inter-trial interval was followed with a random duration between 1 and 2 s (Figure 1A). The delays between two reward were constantly 1 month, which means that the SS reward were always available immediately and the LL reward would be available a month later. The differences between SS and LL reward were adjusted based on individual results of discounting rate estimation procedure for each subject (0.95k, k, and 1.05k). The valences of emotion and SS–LL combinations were randomly presented within a whole block. The IC task consisted of 81 trials, which included 27 conditions (Amount size: small, medial, large × Differences between SS and LL: 0.95k, k, 1.05k × Emotion: negative, neutral and positive).

#### Time Reproduction Task (Figure 1B)

The task was to reproduce the duration of a standard stimulus (1, 2, or 4 s) by pressing “F” on the keyboard after the appearance of a target stimulus. Each trial started with a fixation for 0.5 s followed by a randomly arranged affective picture with one of three valences for 1.5 s. The disappearance of an affective stimulus initiated an inter-stimulus interval (ISI) for 0.3 s which was followed by a standard stimulus (Gray circle with visual angle of 2°) with a duration of 1, 2, or 4 s. During the presentation of the standard stimulus, participants were asked to subjectively estimate the duration of standard stimulus, which would be reproduced later. Another ISI with one of variable durations of 0.4, 0.6, or 0.8 s proceeded. Then, a target stimulus (Gray square with view angle of 2° ) appeared, subjects were required to reproduce the duration of the standard stimuli and pressed “F” on the keyboard when they thought duration of target stimulus was equal to the standard stimulus. Each participant had to finish 45 trials that contained five trials for each standard duration in each emotional condition.

#### Go/NoGo Task (Figure 1C)

Subjects were requested to respond to green cycles (Go stimuli; visual angle of 10° ; frequency, 70%) by immediate button pressing, but to withhold responses to red cycles (NoGo stimuli; visual angle of 10° ; frequency, 30%). 105 Go stimuli and 45 NoGo stimuli (35 Go stimuli and 15 NoGo stimuli for each emotion) were randomly presented on the screen with a duration of 400 ms. After a response, the trial was ended by an ISI.

### Data Analysis

All the responses including the choices as well as the reaction times of each trial were recorded by E-Prime 2.0 (Psychology Software Tools inc. Pittsburgh, PA, USA) and then transferred to Excel and SPSS 17.0 for further analyses.

In the IC task, we calculated the percentages of trials during which SS reward were chosen (SS%) under different emotional contexts. Then, we performed a repeated measures analysis of variance (ANOVA) to examine effects of emotions on the SS%. In order to evaluate the effect of emotion on time perception, we first computed the T_corrected_ score by the following formula: T_corrected_ score = (T_estimated_–T_standard_)/T_standard_ (Noulhiane et al., 2007). This transformation provided information about both extent and direction of the error of temporal estimation. Then we performed a repeated measures analysis of variance (ANOVA) on T_corrected_ scores among different conditions (3 emotions × 3 durations). For performance on the Go/NoGo task, the percentages of commission errors in rare stimuli (NoGo stimuli) trials and percentages of omissions in frequent stimuli (Go stimuli) trials were analyzed by a repeated measures analysis of variances (ANOVA).

Finally, we made an attempt to examine the role of altered time perception induced by emotion in the IC by Spearman’s rho bivariate analysis of correlations between emotional effect on IC (differences of SS% between negative/positive context and neutral context: SS%_negative—SS%_neutral, SS%_positive—SS%_neutral) and emotional effect on time perception (differences of T_corrected_ scores between negative/positive context and neutral context: T_corrected__negative—T_corrected__neutral, T_corrected__positive—T_corrected__neutral).

## Results

### The Effect of Emotion on Inter-Temporal Choice

**FIGURE 2:**
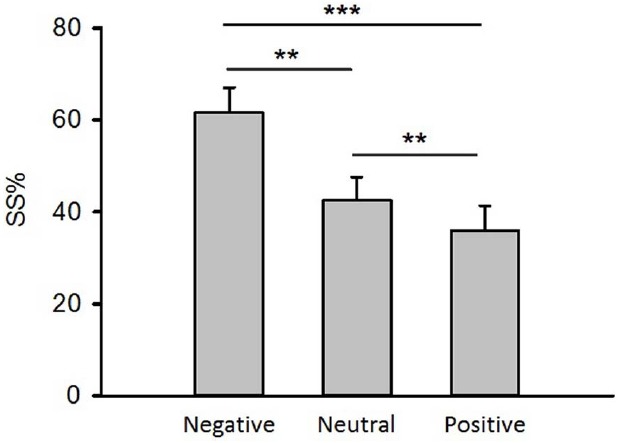
**The effect of emotion on inter-temporal choice.** SS%: the percentage of trials during which smaller-but-sooner reward were chosen. Error bars represent the standard error of means. ***p < 0.001, **p < 0.01, paired *t*-tests.

The repeated measures analysis of variance demonstrated that the main effect of emotion on percentages of trials during which SS reward were chosen (SS%) was significant [Figure 2, *F*(1.11,27.76) = 15.74, *p* < 0.001, ${\text{η}}_{\text{partial}}^{2}$ = 0.39]. Paired *t*-tests showed that compared to neutral priming, negative priming induced much higher SS% [Figure 2, *t*(25) = 3.86, *p* < 0.01] while positive priming generated much lower SS% [Figure 2, *t*(25) = 4.26, *p* < 0.01], and negative priming led to higher SS% than positive priming did [Figure 2, *t*(25) = 3.43, *p* < 0.001]. These data indicated that participants preferred SS reward to delayed reward in negative emotional context, which led to myopic behaviors.

### The Effect of Emotion on Time Perception

**FIGURE 3:**
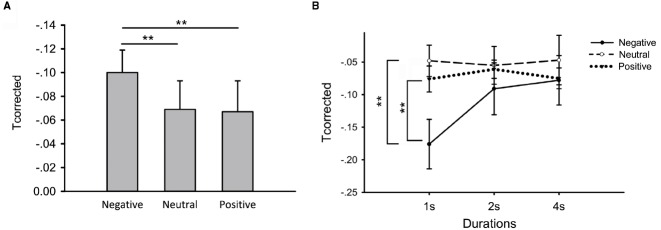
**Differential emotional effects on time perception in the time reproduction task. (A)** Differential effects when durations pooled together; **(B)** Differential effects at different durations. Error bars represent the standard error of means. **p < 0.01 paired *t*-tests.

First, we computed the T_corrected_ scores in different conditions (duration × emotion). Then we examined differential influences of emotion on time reproduction by the repeated measures analysis of variance. We found a significant main effect of emotion [Figure 3A, *F*(2,50) = 3.46, *p* = 0.04, ${\text{η}}_{\text{partial}}^{2}$ = 0.12] and no main effect of duration [*F*(1.50,37.50) = 2.60, *p* > 0.05, ${\text{η}}_{\text{partial}}^{2}$ = 0.09]. However, there was a significant interaction between duration and emotion [*F*(4,100) = 3.84, *p* < 0.01, ${\text{η}}_{\text{partial}}^{2}$ = 0.13]. Thus, we performed further repeated measures analysis of variance and noticed an emotional effect when participants reproduced 1-s-duration [Figure 3B, *F*(2,50) = 6.15, *p* < 0.01, ${\text{η}}_{\text{partial}}^{2}$ = 0.18]. T_corrected_ scores were considerably smaller in negative priming compared with neutral [Figure 3B, *t*(25) = 2.75, *p* < 0.05, paired *t*-test] and positive priming [Figure 3B, *t*(25) = 3.39, *p* < 0.01, paired *t*-test], no difference was found between neutral and positive priming [Figure 3B, *t*(25) = 0.43, *p* > 0.05, paired *t*-test]. There was no main effect of emotion on T_corrected_ scores when participants reproduced 2-s-duration [Figure 3B, *F*(2,50) = 0.07, *p* > 0.05, ${\text{η}}_{\text{partial}}^{2}$ = 0.01] and 4-s-duration [Figure 3B, *F*(2,50) = 1.13, *p* > 0.05, ${\text{η}}_{\text{partial}}^{2}$ = 0.04].

All the above results suggested that negative priming made participants judge time to be longer compared with neutral and positive priming especially in short durations.

### The Effect of Emotion on Response Inhibition

**FIGURE 4:**
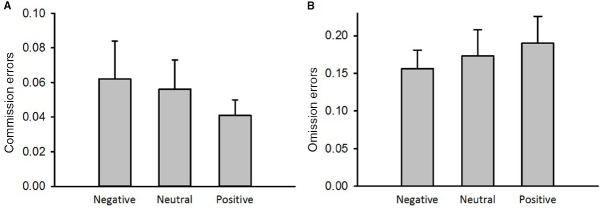
**The emotional effect on performance on the Go/NoGo task. (A)** Commission errors. **(B)** Omission errors. Error bars represent the standard error of means.

The results from a repeated measures analysis of variance indicated no main effect of emotion on the commission errors to NoGo stimuli [Figure 4A, *F*(2,50) = 0.78, *p* > 0.05, ${\text{η}}_{\text{partial}}^{2}$ = 0.03] and omission errors to Go stimuli [Figure 4B, *F*(2,50) = 1.49, *p* > 0.05, ${\text{η}}_{\text{partial}}^{2}$ = 0.06]. Therefore, response inhibition measured by a Go/NoGo task was not affected by emotion priming.

### The Correlations Between Inter-Temporal Choice and Time Perception

**FIGURE 5:**
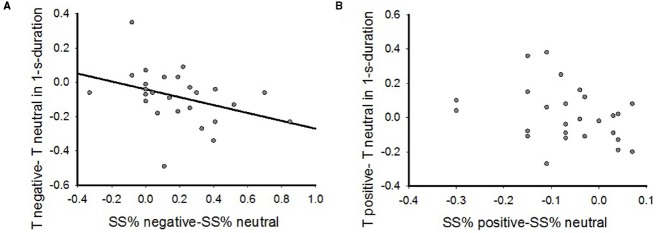
**The correlations between emotional effects on inter-temporal choice and time perception by Spearman’s rho bivariate analysis. (A)** The correlations in negative emotional context in 1-s reproduction task; **(B)** The correlations in the positive emotional context in 1-s reproduction task.

In order to examine the interaction between time perception and IC in the emotional contexts, we correlated the emotional priming effect on time perception (T_corrected__negative–T_corrected__neutral, T_corrected__positive–T_corrected__neutral) to the emotional priming effect on IC (SS%_negative—SS% neutral, SS%_positive—SS%_neutral) by the spearman’s rho bivariate analysis. The results showed that the altered time perception by negative priming relative to neutral priming was negatively correlated to the change of IC when participants performed 1-s reproduction task (Figure 5A, *r* = (–0.45, *p* = 0.02), while there was no significant correlation in positive emotional context (Figure 5B, *r* = –0.35, *p* > 0.05). The correlation was also absent in both 2-s reproduction task (negative: *r* = 0.09, *p* > 0.05; positive: *r* = –0.15, *p* > 0.05) and 4-s reproduction task (negative: *r* = 0.03, *p* > 0.05; positive: *r* = –0.03, *p* > 0.05). Therefore, these results indicated myopic decision making induced by negative emotional priming could be associated with altered time perception.

## Discussion

In the present study, we found that temporary negative priming induced by affective pictures in a trial-to-trial paradigm resulted in much higher percentage of trials during which SS reward were chosen compared with positive priming and neutral priming. While reproducing a shorter duration of time, subjects tended to perceive it as a much longer time in a negative emotional context. In the other words, humans might overestimate the subjective experience of time in a negative state. Moreover, correlation analyses indicated that altered time perception was negatively correlated with the changes of IC, while the emotional effect did not influence performance including commission errors and omission errors in a Go/NoGo task. Therefore, our findings indicated that myopic decisions induced by negative emotions could be associated with altered time perception but not response inhibition.

### Negative Emotion and Myopic Behavior

Using a trial-to-trial paradigm, we found that participants preferred SS reward to LL ones in the temporary negative context compared with positive and neutral contexts, which indicates that negative emotion makes people be myopic in the decision making task. These results were consistent with previous studies in which stable emotional contexts were induced through block of task or whole task (Augustine and Larsen, 2011; Li et al., 2011; Liu et al., 2013). However, Luo et al. (2014) reported that temporally negative priming by facial expressions resulted in much higher percentage of LL choices, which means negative priming makes people more far-sighted (Luo et al., 2014). The experimental procedures in our present study were pretty similar with Luo’s study except emotional materials, the IAPS pictures versus facial expressions. However, in our unpublished study, we induced emotional contexts by facial expressions in the same paradigm. We also found that negative context resulted in myopic behaviors in the IC task, which was consistent with findings in our present study. Taken together, we propose that people may usually make myopic decision under negative context independent of emotional material. In Luo et al’s study, participants were asked to perform a facial memory task while performing IC task, which would increase cognitive load. Growing evidence has shown that there is significant link between cognitive load and delay discounting. For example, Hinson et al. (2003) reported that discounting rate was larger under higher working memory load compared to lower working memory load. Similarly, another study revealed significant negative correlation between memory span and discounting rates (Shamosh et al., 2008). Therefore, we think that different levels of cognitive load while performing IC task might be one of the main factors resulting in the inconsistent results between Luo et al’s and ours. In addition, humans usually maximize profits when they attempt to make decisions among alternatives. Based on earlier findings, gratitude impelled an attempt to increase long-term gains in social networks at the expense of short-term monetary costs (DeSteno, 2009). In this regard, facial expressions might induce a kind of inter-personal emotions. Positive facial expressions might led to a preference for SS reward because SS reward can bring some potential values, such as inter-personal network. Therefore, the different level of social emotions induced by IAPS pictures and facial expression pictures might also result in the inconsistent findings between Luo et al’s and ours.

One limitation in our present study was that we did not match the arousal in different emotional conditions. However, the myopic decisions induced by negative emotion in our present study could not result from the arousal level of the emotions because we did not find a significant correlation between arousal level of emotional pictures and SS% in the IC task (Spearman rho, all *r*s < 0.3, all *p*s > 0.05).

### Time Perception and Inter-Temporal Choice

Time perception has consistently been considered to be a crucial factor in the inter-temporal decision making (Wittmann and Paulus, 2008). Presume a person subjectively considers a duration of time is too long, he will prefer immediate reward because the delayed ones, though they are much larger, are not worth waiting for. Therefore, the sense of time should theoretically change the performance on inter-temporal decisions. A growing body of evidence has consistently revealed an indirect relationship between time perception and IC. A lot of mood and psychological disorders displayed distorted subjective time perception and impaired inter-temporal decisions. Bschor et al. (2004) found that depressive patients tended to underestimate durations in temporal production (35 and 90 s) and verbal estimation tasks (109 s) compared with non-depressive patients (Bschor et al., 2004). However, schizophrenia patients often displayed overestimation of time (Lee et al., 2009; Bonnot et al., 2011; Papageorgiou et al., 2013). Meanwhile, other evidence revealed that schizophrenic individuals preferred immediate reward even with a small magnitude (Heerey et al., 2007; Weller et al., 2014). Nevertheless, the direct relationship between altered time perception and IC has not been examined in previous studies.

Previous evidence has shown that negative emotional state induced by emotional sounds (Noulhiane et al., 2007), IAPS pictures (Gil and Droit-Volet, 2012), and facial expressions (Droit-Volet et al., 2004; Bar-Haim et al., 2010) led to much longer time estimations compared with neutral or positive states. In a recent study, Buetti and Lleras (2012) demonstrated that IAPS negative pictures were perceived as lasting longer than positive pictures when both spider-fearful participants and non-anxious participants performed a time bisection task (Buetti and Lleras, 2012). The negative emotion-related lengthening effect in our present study was consistent with other findings. We noticed that compared with positive and neutral emotional primings, negative priming resulted in much lower T_corrected_ scores, which suggests that negative priming leads to overestimation of time, especially in the short time reproduction task. Meanwhile, SS% in negative context was much higher than that in the other two emotional contexts. More importantly, the differences of T_corrected_ scores between negative and neutral primings in short condition were significantly correlated to the differences of SS% between those emotional primings, while no significant correlation was found in the positive emotional context. All these findings strongly supported the idea that myopic decision making behaviors induced by temporal negative priming could be associated with the overestimation of time in the negative context.

Interestingly, we found that the correlation between time estimation and IC existed only in short duration trials (1 s) rather than longer duration trials (2 or 4 s). In a recent study, it was found that depressive patients were less able to discriminate between two longer durations (>1000 ms) than control group in a time discrimination task. But two groups were equally able to discriminate shorter intervals (<300 ms; Msetfi et al., 2012). Thus, it is reasonable to hypothesize that mild depression affects the ability to maintain attention to elapsing duration but not pacemaker speed in the internal clock model. Cumulative evidence has revealed that the processing of sub- and supra-second durations relies on different cognitive mechanisms. The processing of short durations appears to be automatic, whereas long durations seem to require not only to hold temporal information in working memory, but also to continue to concentrate their attention on the passage of time until the end of the stimulus (Lewis and Miall, 2003, 2006). With regard to neural basis of time perception, dissociable neural networks have been associated with estimation of subsecond and suprasecond durations. The subsecond duration discrimination is related to activation in bilateral anterior cerebellum, whereas the suprasecond duration estimation is associated with activation in the inferior parietal lobule (Hayashi et al., 2014). These results support the idea that subsecond durations are processed in the motor system, whereas suprasecond durations are processed in the parietal cortex by utilizing the capacity of attention and working memory to keep track of time. Moreover, a recent study showed that activations in the medial/inferior frontal cortex and the inferior parietal cortex were correlated with underestimation of time in the time reproduction task. Meanwhile, the activations in these regions were also associated with the less pronounced the future perspective measured by the Zimbardo time perspective inventory (Wittmann et al., 2011). In our present study, the duation of time reproduction (<4 s) is much shorter than delay duration (1 month) in the IC task. It is impossible to ask participants to reproduce a longer duration (such as couple of hours, days) in the lab. However, we could measure the subjective time perception of a longer duration (i.e., 1 month in our study) by the Zimbardo time perspective inventory in the future study and explore the interaction between time perception and IC. Participants are usually asked to make a number of choices between smaller immediate reward and larger reward weeks, months, or years in the future in most of typical human studies on IC. However, shorter delay duration in the scale of second has also found in IC studies on animals (Kim et al., 2008; Blanchard and Hayden, 2015) and humans (Schweighofer et al., 2006; Luhmann et al., 2008). In order to maximize total gain, people would make delayed reward choices based on hyperbolic or exponential discounting in different situations. For example, humans adopted exponential discounting when they performed in a reward decision task with temporal constraints in the scale of second (Schweighofer et al., 2006), while other behavioral studies in humans well favored hyperbolic discounting (Kirby and Petry, 2004; Kim et al., 2012; Luo et al., 2014). Therefore, it would be interesting to explore whether emotional effects on IC are the same or not when participants make choices in the IC task in different situations in the future study, such as in the situation of shorter (i.e., seconds) and longer (i.e., months) pursuit time. Whatever the mechanisms are, intervention might eventually be able to alter directly the timing system, which in turn could profoundly affect the way individuals process delayed reward and structure behavior toward health-promoting actions (Wittmann and Paulus, 2008).

### Response Inhibition and Inter-Temporal Choice

Impulsivity is a multidimensional concept that incorporates failure of response inhibition, rapid processing of information, novelty seeking, and inability to delay gratification. Most attempts to measure impulsivity rely on psychometric self-report trait measures, such as Barratt Impulsiveness Scale -11, Eysenck Impulsiveness Scale and other questionnaires. Petry (2002) demonstrated that substance abusers displayed significant higher impulsivity scores by Eysenck Impulsiveness Scale and discounted hypothetical monetary reward at a higher rate than healthy controls. Moreover, the Eysenck impulsivity scores were positively correlated with k values (Petry, 2002). Another element of impulsivity, response inhibition is often measured by a Go/NoGo task, which requires speedy motor responses to one type of stimuli and inhibition of responses to another type of stimuli (Goya-Maldonado et al., 2010). In the present study, we used a Go/NoGo task to examine response inhibition (or motor impulsivity) in different emotional contexts. We found that negative emotional context lead to myopic behaviors in the IC task, while it did not result in significant change of commission/omission errors in the Go/NoGo task. Further, the Spearman’s rho bivariate analysis did not show correlation between performance of Go/NoGo task and IC in negative context (*r* = –0.21, *p* = 0.30) or positive context (*r* = –0.19, *p* = 0.35). It seems that motor impulsivity plays a little role in emotional effect on IC. Some other studies can also support our explanation. For example, Coffey et al. (2011) reported that borderline personality disorders with substance using disorders displayed many more errors in NoGo signals compared with matched controls, meanwhile they also displayed higher discount rates in delay discounting tasks. Nevertheless, there was no correlation between poor response inhibition and impaired IC. In contrast, the self-reported impulsivity by Barratt Impulsiveness Scale -11 was significantly correlated to delay discounting task (Coffey et al., 2011). Therefore, our findings and previous evidence suggested that impulsivity measured by different psychological tools could have variable interactions between impulsivity and IC. A recent study showed that the mechanisms underlying the motor subtype of impulsivity were dissociable from the temporal and reflection subtypes (Caswell et al., 2013). Therefore, the exact of cognitive and neural networks underlying different subtypes impulsivity need to be elucidated in the future. Meanwhile, since we did not measure impulsivity using a set of questionnaires or other tools, it is hard to illustrate whether other kinds of impulsivities play a function in IC. Therefore, future studies should focus on whether other subtypes of impulsivity play roles in the modulation of emotion in IC.

Impulsivity is multidetermined. The subconstruct of urgency represents individual differences in the tendency to engage in ill-considered actions when experiencing intense emotion (Cyders and Smith, 2008). It has been correlated with striatal dopamine release, activation in the medial prefrontal cortex (Weiland et al., 2014) and amygdala (Cyders and Smith, 2008). Urgency scores in schizophrenia were correlated with both reduced cortical thickness in ventral prefrontal regions and reduced resting-state functional connectivity (Hoptman et al., 2014). In a recent study, Kraplin et al. (2014) reported that PGs displayed much higher level of urgency and greater discounting rate in the delay discounting task compared to normal controls. Moreover, the discounting rate was positively correlated with the level of urgency (Kraplin et al., 2014). Based on these findings, urgency induced by negative emotion could be one possibility to explain the emotional effect on IC task in our present study. Therefore, we should measure the multidimensional impulsivity by the UPPS impulsive behavioral scale and explore the relationships between myopic decisions and subconstructs of impulsivity (even the activations in certain brain areas, such as amygdala, anterior insula, inferior/medial frontal cortex) in the future study.

## Conclusion

The temporary negative priming resulted in the preference for SS choices in the task of IC. Such myopic behavior was probably related to alteration of time perception by negative emotion and not to response inhibition.

### Conflict of Interest Statement

The authors declare that the research was conducted in the absence of any commercial or financial relationships that could be construed as a potential conflict of interest.

## References

[B1] Angrilli A. Cherubini P. Pavese A. Mantredini S. (1997). The influence of affective factors on time perception. Percept. Psychophys. 59, 972–982. 10.3758/BF03205512 9270369

[B2] Asutay E. Vastfjall D. (2012). Perception of loudness is influenced by emotion. PLoS ONE 7:e38660. 10.1371/journal.pone.0038660 22685594 PMC3369848

[B3] Augustine A. A. Larsen R. J. (2011). Affect regulation and temporal discounting: interactions between primed, state, and trait affect. Emotion 11, 403–412. 10.1037/a0021777 21500908

[B4] Bar-Haim Y. Kerem A. Lamy D. Zakay D. (2010). When time slows down: the influence of threat on time perception in anxiety. Cogn. Emot. 24, 255–263 10.1080/02699930903387603

[B5] Billieux J. Lagrange G. Van Der Linden M. Lancon C. Adida M. Jeanningros R. (2012). Investigation of impulsivity in a sample of treatment-seeking pathological gamblers: a multidimensional perspective. Psychiatry Res. 198, 291–296. 10.1016/j.psychres.2012.01.001 22421073

[B6] Blanchard T. C. Hayden B. Y. (2015). Monkeys are more patient in a foraging task than in a standard intertemporal choice task. PLoS ONE 10:e0117057. 10.1371/journal.pone.0117057 25671436 PMC4324901

[B7] Bonnot O. De Montalembert M. Kermarrec S. Botbol M. Walter M. Coulon N. (2011). Are impairments of time perception in schizophrenia a neglected phenomenon? J. Physiol. Paris 105, 164–169. 10.1016/j.jphysparis.2011.07.006 21803155

[B8] Brosch T. Scherer K. R. Grandjean D. Sander D. (2013). The impact of emotion on perception, attention, memory, and decision-making. Swiss Med. Wkly 143, w13786. 10.4414/smw.2013.13786 23740562

[B9] Brosch T. Van Bavel J. J. (2012). The flexibility of emotional attention: accessible social identities guide rapid attentional orienting. Cognition 125, 309–316. 10.1016/j.cognition.2012.07.007 22863414

[B10] Bschor T. Ising M. Bauer M. Lewitzka U. Skerstupeit M. Muller-Oerlinghausen B. (2004). Time experience and time judgment in major depression, mania and healthy subjects. A controlled study of 93 subjects. Acta Psychiatr. Scand. 109, 222–229. 10.1046/j.0001-690X.2003.00244.x 14984395

[B11] Buetti S. Lleras A. (2012). Perceiving control over aversive and fearful events can alter how we experience those events: an investigation of time perception in spider-fearful individuals. Front. Psychol. 3:337. 10.3389/fpsyg.2012.00337 23060824 PMC3444055

[B12] Caswell A. J. Morgan M. J. Duka T. (2013). Inhibitory Control Contributes to “Motor”—but not “Cognitive”—impulsivity. Exp. Psychol. 60, 324–334. 10.1027/1618-3169/A000202 23628696

[B13] Coffey S. F. Gudleski G. D. Saladin M. E. Brady K. T. (2003). Impulsivity and rapid discounting of delayed hypothetical rewards in cocaine-dependent individuals. Exp. Clin. Psychopharmacol. 11, 18–25. 10.1037/1064-1297.11.1.18 12622340

[B14] Coffey S. F. Schumacher J. A. Baschnagel J. S. Hawk L. W. Holloman G. (2011). Impulsivity and risk-taking in borderline personality disorder with and without substance use disorders. Personal. Disord. 2, 128–141. 10.1037/a0020574 22448732

[B15] Cyders M. A. Smith G. T. (2008). Emotion-based dispositions to rash action: positive and negative urgency. Psychol. Bull. 134, 807–828. 10.1037/a0013341 18954158 PMC2705930

[B16] Demurie E. Roeyers H. Baeyens D. Sonuga-Barke E. (2013). Domain-general and domain-specific aspects of temporal discounting in children with ADHD and autism spectrum disorders (ASD): a proof of concept study. Res. Dev. Disabil. 34, 1870–1880. 10.1016/j.ridd.2013.03.011 23578902

[B17] DeSteno D. (2009). Social emotions and intertemporal choice “Hot” mechanisms for building social and economic capital. Curr. Dir. Psychol. Sci. 18, 280–284 10.1111/j.1467-8721.2009.01652.x

[B18] Dixon M. R. Marley J. Jacobs E. A. (2003). Delay discounting by pathological gamblers. J. Appl. Behav. Anal. 36, 449–458. 10.1901/jaba.2003.36-449 14768665 PMC1284461

[B19] Droit-Volet S. (2013). Time perception, emotions and mood disorders. J. Physiol. Paris 107, 255–264. 10.1016/j.jphysparis.2013.03.005 23542546

[B20] Droit-Volet S. Brunot S. Niedenthal P. M. (2004). Perception of the duration of emotional events. Cogn. Emot. 18, 849–858 10.1080/02699930341000194

[B21] Droit-Volet S. Fayolle S. L. Gil S. (2011). Emotion and time perception: effects of film-induced mood. Front. Integr. Neurosci. 5:33. 10.3389/fnint.2011.00033 21886610 PMC3152725

[B22] Gil S. Droit-Volet S. (2012). Emotional time distortions: the fundamental role of arousal. Cogn. Emot. 26, 847–862. 10.1080/02699931.2011.625401 22296278

[B23] Goya-Maldonado R. Walther S. Simon J. Stippich C. Weisbrod M. Kaiser S. (2010). Motor impulsivity and the ventrolateral prefrontal cortex. Psychiatry Res. 183, 89–91. 10.1016/j.pscychresns.2010.04.006 20542670

[B24] Green L. Fry A. F. Myerson J. (1994). Discounting of delayed rewards—a life-span comparison. Psychol. Sci. 5, 33–36 10.1111/j.1467-9280.1994.tb00610.x

[B25] Green L. Myerson J. Lichtman D. Rosen S. Fry A. (1996). Temporal discounting in choice between delayed rewards: the role of age and income. Psychol. Aging 11, 79–84. 10.1037/0882-7974.11.1.79 8726373

[B26] Hayashi M. J. Kanai R. Tanabe H. C. Yoshida Y. Carlson S. Walsh V. (2013). Interaction of numerosity and time in prefrontal and parietal cortex. J. Neurosci. 33, 883–893. 10.1523/JNEUROSCI.6257-11.2013 23325227 PMC6704870

[B27] Hayashi M. J. Kantele M. Walsh V. Carlson S. Kanai R. (2014). Dissociable neuroanatomical correlates of subsecond and suprasecond time perception. J. Cogn. Neurosci. 26, 1685–1693. 10.1162/jocn_a_00580 24456398

[B28] Heerey E. A. Robinson B. M. Mcmahon R. P. Gold J. M. (2007). Delay discounting in schizophrenia. Cogn. Neuropsychiatry 12, 213–221. 10.1080/13546800601005900 17453902 PMC3746343

[B29] Hinson J. M. Jameson T. L. Whitney P. (2003). Impulsive decision making and working memory. J. Exp. Psychol. Learn. Mem. Cogn. 29, 298–306. 10.1037/0278-7393.29.2.298 12696817

[B30] Hoptman M. J. Antonius D. Mauro C. J. Parker E. M. Javitt D. C. (2014). Cortical thinning, functional connectivity, and mood-related impulsivity in schizophrenia: relationship to aggressive attitudes and behavior. Am. J. Psychiatry 171, 939–948. 10.1176/appi.ajp.2014.13111553 25073506 PMC4178944

[B31] Kim B. K. Zauberman G. (2009). Perception of anticipatory time in temporal discounting. J. Neurosci. Psychol. Econ. 2, 91–101 10.1037/a0017686

[B32] Kim B. Sung Y. S. Mcclure S. M. (2012). The neural basis of cultural differences in delay discounting. Philos. Trans. R. Soc. Lond. B Biol. Sci. 367, 650–656. 10.1098/rstb.2011.0292 22271781 PMC3260846

[B33] Kim S. Hwang J. Lee D. (2008). Prefrontal coding of temporally discounted values during intertemporal choice. Neuron 59, 161–172. 10.1016/j.neuron.2008.05.010 18614037 PMC2593737

[B34] Kirby K. N. Petry N. M. (2004). Heroin and cocaine abusers have higher discount rates for delayed rewards than alcoholics or non-drug-using controls. Addiction 99, 461–471. 10.1111/j.1360-0443.2003.00669.x 15049746

[B35] Kirby K. N. Petry N. M. Bickel W. K. (1999). Heroin addicts have higher discount rates for delayed rewards than non-drug-using controls. J. Exp. Psychol. Gen. 128, 78–87. 10.1037/0096-3445.128.1.78 10100392

[B36] Kraplin A. Dshemuchadse M. Behrendt S. Scherbaum S. Goschke T. Buhringer G. (2014). Dysfunctional decision-making in pathological gambling: pattern specificity and the role of impulsivity. Psychiatry Res. 215, 675–682. 10.1016/j.psychres.2013.12.041 24434041

[B37] Lang P. J. Bradley M. M. Cuthbert B. N. (2005). International Affective Picture System (IAPS): Affective Ratings of Pictures and Instruction Manual. Gainesville, FL: NIMH, Center for the Study of Emotion and Attention.

[B38] Lee K. H. Bhaker R. S. Mysore A. Parks R. W. Birkett P. B. Woodruff P. W. (2009). Time perception and its neuropsychological correlates in patients with schizophrenia and in healthy volunteers. Psychiatry Res. 166, 174–183. 10.1016/j.psychres.2008.03.004 19278734

[B39] Lewis P. A. Miall R. C. (2003). Distinct systems for automatic and cognitively controlled time measurement: evidence from neuroimaging. Curr. Opin. Neurobiol. 13, 250–255. 10.1016/S0959-4388(03)00036-9 12744981

[B40] Lewis P. A. Miall R. C. (2006). Remembering the time: a continuous clock. Trends Cogn. Sci. 10, 401–406. 10.1016/j.tics.2006.07.006 16899395

[B41] Li J. Z. Li S. Liu H. (2011). How has the wenchuan earthquake influenced people’s intertemporal choices? J. Appl. Soc. Psychol. 41, 2739–2752 10.1111/j.1559-1816.2011.00847.x

[B42] Liu L. Feng T. Chen J. Li H. (2013). The value of emotion: how does episodic prospection modulate delay discounting? PLoS ONE 8:e81717. 10.1371/journal.pone.0081717 24312341 PMC3842935

[B43] Lucci C. R. (2013). Time, self, and intertemporal choice. Front. Neurosci. 7:40. 10.3389/fnins.2013.00040 23750125 PMC3664308

[B44] Luhmann C. C. Chun M. M. Yi D. J. Lee D. Wang X. J. (2008). Neural dissociation of delay and uncertainty in intertemporal choice. J. Neurosci. 28, 14459–14466. 10.1523/JNEUROSCI.5058-08.2008 19118180 PMC2742332

[B45] Luo S. Ainslie G. Monterosso J. (2014). The behavioral and neural effect of emotional primes on intertemporal decisions. Soc. Cogn. Affect. Neurosci. 9, 283–291. 10.1093/scan/nss132 23160811 PMC3980799

[B46] Martin L. N. Delgado M. R. (2011). The influence of emotion regulation on decision-making under risk. J. Cogn. Neurosci. 23, 2569–2581. 10.1162/jocn.2011.21618 21254801 PMC3164848

[B47] Mazur J. E. (1987). “An adjusting procedure for studying delayed reinforcement,” in The Effect of Delay and of Intervening Events on Reinforcement Value, eds Commons M. L. Mazur J. E. Nevin J. A. Rachlin H. (Hillsdale, NJ: Lawrence Erlbaum Associates, Inc), 55–73.

[B48] McClure S. M. Laibson D. I. Loewenstein G. Cohen J. D. (2004). Separate neural systems value immediate and delayed monetary rewards. Science 306, 503–507. 10.1126/science.1100907 15486304

[B49] Milenkova M. Mohammadi B. Kollewe K. Schrader C. Fellbrich A. Wittfoth M. (2011). Intertemporal choice in Parkinson’s disease. Mov. Disord. 26, 2004–2010. 10.1002/mds.23756 21567457

[B50] Msetfi R. M. Murphy R. A. Kornbrot D. E. (2012). The effect of mild depression on time discrimination. Q. J. Exp. Psychol. 65, 632–645. 10.1080/17470218.2011.608908 22313021

[B51] Noulhiane M. Mella N. Samson S. Ragot R. Pouthas V. (2007). How emotional auditory stimuli modulate time perception. Emotion 7, 697–704. 10.1037/1528-3542.7.4.697 18039036

[B52] Papageorgiou C. Karanasiou I. S. Kapsali F. Stachtea X. Kyprianou M. Tsianaka E. I. (2013). Temporal processing dysfunction in schizophrenia as measured by time interval discrimination and tempo reproduction tasks. Prog. Neuropsychopharmacol. Biol. Psychiatry 40, 173–179. 10.1016/j.pnpbp.2012.07.017 23367507

[B53] Peters J. (2011). The role of the medial orbitofrontal cortex in intertemporal choice: prospection or valuation? J. Neurosci. 31, 5889–5890. 10.1523/JNEUROSCI.0268-11.2011 21508212 PMC6632962

[B54] Petry N. M. (2002). Discounting of delayed rewards in substance abusers: relationship to antisocial personality disorder. Psychopharmacology 162, 425–432. 10.1007/s00213-002-1115-1 12172697

[B55] Power Y. Goodyear B. Crockford D. (2012). Neural correlates of pathological gamblers preference for immediate rewards during the Iowa gambling task: an fMRI study. J. Gambl. Stud. 28, 623–636. 10.1007/s10899-011-9278-5 22037936

[B56] Rimmele U. Davachi L. Petrov R. Dougal S. Phelps E. A. (2011). Emotion enhances the subjective feeling of remembering, despite lower accuracy for contextual details. Emotion 11, 553–562. 10.1037/a0024246 21668106 PMC3864593

[B57] Schulz K. P. Fan J. Magidina O. Marks D. J. Hahn B. Halperin J. M. (2007). Does the emotional go/no-go task really measure behavioral inhibition? Convergence with measures on a non-emotional analog. Arch. Clin. Neuropsychol. 22, 151–160. 10.1016/j.acn.2006.12.001 17207962 PMC2562664

[B58] Schweighofer N. Shishida K. Han C. E. Okamoto Y. Tanaka S. C. Yamawaki S. (2006). Humans can adopt optimal discounting strategy under real-time constraints. PLoS Comput. Biol. 2:e152. 10.1371/journal.pcbi.0020152 17096592 PMC1635539

[B59] Shamosh N. A. Deyoung C. G. Green A. E. Reis D. L. Johnson M. R. Conway A. R. A. (2008). Individual differences in delay discounting: relation to intelligence, working memory, and anterior prefrontal cortex. Psychol. Sci. 19, 904–911. 10.1111/j.1467-9280.2008.02175.x 18947356

[B60] Takahashi T. (2005). Loss of self-control in intertemporal choice may be attributable to logarithmic time-perception. Med. Hypotheses 65, 691–693. 10.1016/j.mehy.2005.04.040 15990243

[B61] Takahashi T. Oono H. Inoue T. Boku S. Kako Y. Kitaichi Y. (2008). Depressive patients are more impulsive and inconsistent in intertemporal choice behavior for monetary gain and loss than healthy subjects–an analysis based on Tsallis’ statistics. Neuro Endocrinol. Lett. 29, 351–358. 18580849

[B62] Weatherly J. N. (2012). The association between delay discounting and schizotypal personality characteristics in a nonclinical sample. Psychol. Rec. 62, 529–540.

[B63] Weiland B. J. Heitzeg M. M. Zald D. Cummiford C. Love T. Zucker R. A. (2014). Relationship between impulsivity, prefrontal anticipatory activation, and striatal dopamine release during rewarded task performance. Psychiatry Res. 223, 244–252. 10.1016/j.pscychresns.2014.05.015 24969539 PMC4136473

[B64] Weller R. E. Avsar K. B. Cox J. E. Reid M. A. White D. M. Lahti A. C. (2014). Delay discounting and task performance consistency in patients with schizophrenia. Psychiatry Res. 215, 286–293. 10.1016/j.psychres.2013.11.013 24388727 PMC4388189

[B65] Wittmann M. Paulus M. P. (2008). Decision making, impulsivity and time perception. Trends Cogn. Sci. 12, 7–12. 10.1016/j.tics.2007.10.004 18042423

[B66] Wittmann M. Simmons A. N. Flagan T. Lane S. D. Wackermann J. Paulus M. P. (2011). Neural substrates of time perception and impulsivity. Brain Res. 1406, 43–58. 10.1016/j.brainres.2011.06.048 21763642 PMC3145046

